# Surface Topography and Ultrastructure of the Spectacular Cells in the Eyes of Land and Sea Snakes (Squamata, Reptilia): Functional Adaptations of Micro‐Ornamentation

**DOI:** 10.1002/jmor.70084

**Published:** 2025-09-10

**Authors:** H. Barry Collin, Myoung Hoon Ha, Alizee Wagner, Megan Folwell, Nathan Dunstan, Jenna Crowe‐Riddell, Shaun P. Collin

**Affiliations:** ^1^ Department of Optometry and Vision Science University of New South Wales Kensington New South Wales Australia; ^2^ School of Agriculture, Biomedicine and Environment La Trobe University Melbourne Victoria Australia; ^3^ School of Biological Sciences The University of Adelaide Adelaide South Australia Australia; ^4^ Venom Supplies Tanunda South Australia Australia; ^5^ Max Planck Queensland Centre (MPQC) for the Materials Science of Extracellular Matrices Queensland University of Technology Brisbane Queensland Australia

**Keywords:** microholes, micropits, microridges, nanostructures, scales, spectacle

## Abstract

Although the surface micro‐ornamentation of the scales within the skin of snakes has been the subject of many previous studies, there has been little work done on the spectacle, a protective (keratinised) goggle separated from the underlying cornea by a sub‐spectacular space. The surface ultrastructure of the “Oberhäutchen” of the spectacle is examined in nine species of snakes (five aquatic and four terrestrial) using light and electron microscopy, micro‐computed tomography and gel‐based profilometry. Significant topographic differences in cell size (increases of between 5.4% and 165% in the periphery), shape (central pentagonal/hexagonal to long peripheral) and density (2579–10,391 cells/mm^2^ in the centre vs. 2315–4291 cells/mm^2^ in the periphery) are revealed. Small indentations in the surface (micropits) and/or microholes in the cell membrane decorate the epithelial surface of all species, which also show a centre‐to‐periphery gradient in diameter (42.39–120.55 nm in the centre vs*.* 63.76–182.60 nm in the periphery). Microridges are found on the superficial cells of the spectacle of only one species (the terrestrial Cantil Viper, *Agkistrodon bilineatus*) with straight, parallel ridges in the centre (138.4 ± 28.2 nm wide) and a more complex pattern of ridges (143.1 ± 19.1 nm wide) in the periphery. The micro‐ornamentation of the spectacle in both land and sea snakes is compared with those found over the body scales and discussed with respect to a range of potential functions, while still allowing a clear optical pathway for vision.

## Graphical abstract

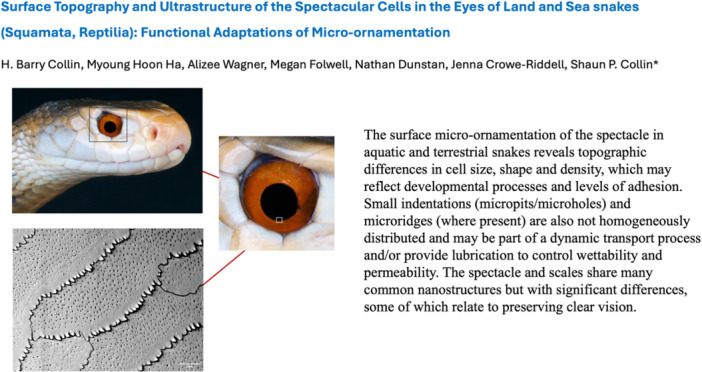

## 1 Introduction

The ocular covering of the eyes of both land and sea snakes has been described as a tertiary spectacle by Walls (1942) who considered this to be a goggle that is composed of tissue completely separate from the underlying eye. The origin of the spectacle stems from the fusion of embryonic tissue that would have otherwise formed eyelids (primarily the lower lid according to Walls 1942) (Schwartz‐Karsten 1933; Neher 1935; Bellairs and Boyd 1947) to form a protective layer that has subsequently become transparent and, in land snakes, has assumed the refractive power previously performed by the cornea (Walls 1942; Duke‐Elder 1958). The anatomy of the spectacle is similar to the superficial regions of the skin or integument, with which it is continuous.

The spectacle is a fixed and immobile “Brille” that is protective, while still allowing the underlying eye to freely rotate and monitor its environment, and is separated from the underlying cornea by a subspectacular space or a “blind intraconjunctival space derived from the cul‐de‐sac of its lidded ancestor” (Walls 1942), filled with an oily lubricant secreted by the Harderian gland (Schwab 2011). Since many snakes are fossorial and actively interrogate their environment, which inevitably causes damage (grazes) and accumulates debris, the spectacle has become relatively thick and the outer layers keratinised (Da Silva et al. 2016). Consequently, a process of ecdysis (or shedding) of these keratinised layers has evolved due to the need to maintain ocular transparency for clear vision in addition to allowing the body (including the eyes) to grow in size throughout life, a process recently termed indeterminate growth (Maderson 1965; Tusler et al. 2015; Cazalot et al. 2015; Hariharan et al. 2016).

The spectacle scale or “stratum corneum” of snakes is also vascularised, containing blood vessels that undergo cycles of dilation and constriction, but maintain a constricted state to minimise visual disturbance when the snake is hunting or faced with a visual threat (Mead 1976; van Doorn and Sivak 2013). Beneath the spectacle, and separated by the sub‐spectacular space, lies the cornea, which, like the spectacle, varies in thickness and morphology according to body length (and eye size), habitat and daily activity patterns (El‐Bakry 2011; Da Silva et al. 2017, 2019). Although snakes possess a series of interfaces within their anterior eye (spectacle, sub‐spectacular space and cornea), transparency is maintained for visual tasks and accommodation is mediated by the antero‐posterior movement of a non‐deformable, spherical lens by peripheral irideal muscles that place pressure on the vitreous (Sivak 1975), a mechanism with some traits in common with their aquatic ancestors (Caprette et al. 2004).

The structure and function of the snake spectacle have received some attention. It is comprised of a stroma composed primarily of collagen fibres bounded by an epithelium with its basement membrane on the anterior (outer) surface and an endothelium with its basement membrane on the posterior surface (Lauridsen et al. 2014; Da Silva et al. 2014, 2016). The outer layers of the epithelial cells regularly become keratinised and, in the skin, are known as the Oberhäutchen (Arrigo et al. 2019; Gower 2003). The shedding cycle, which occurs between 4 and 12 times per year and is triggered by the release of hormones, is an active process of growth, proliferation, differentiation and shedding, where many of the epithelial layers are duplicated, generating outer and inner epidermal zones, which separate during ecdysis, where the outer epidermal zone is lost, leaving the inner epidermal zone (Maderson 1965; Chang et al. 2009; Lauridsen et al. 2014; Lillywhite and Menon 2019). It is presumed that a similar process occurs for the snake spectacle but this has not yet been examined. The “Oberhäutchen of the spectacle” is highly transmissive to light including from the far red to ultraviolet, but in most species blocking the mid to far UV‐A and UV‐B components of the spectrum (van Doorn and Sivak 2015) only transmitting the wavelengths in the UV‐A range that match the specific spectral absorbance characteristics of the retinal opsins (Simões et al. 2016). The eyes of some species also contain a yellow pigment within either their spectacle or lens, which also acts as a barrier to parts of the UV spectrum and increases retinal image contrast (Hart et al. 2012). Spectral transmission is known to be dependent on the chemical composition of the β‐keratin found in the snake spectacle (van Doorn et al. 2014; van Doorn and Sivak 2015), which presumably will vary according to concentration and stage of ecdysis.

Previous studies that have examined the thickness of the snake spectacle include a comparison of 44 species from 14 families using optical coherence tomography (Da Silva et al. 2017), where, in broad terms, arboreal and terrestrial snakes have thin spectacles and fossorial and aquatic snakes possess thick spectacles, presumably an adaptive trade‐off to reduce the risk of damage, while optimising visual acuity. The dermis of most sea snakes has also been found to be 50% thicker to protect against physical injury and resist epibionts (Shine et al. 2019). Da Silva et al. (2015) have examined the histology of the spectacle in 60 species of snakes from five families with respect to inflammatory responses to pathological insults. The structure of the spectacle has been examined using light microscopy (Walls 1942; Da Silva et al. 2014, 2016; Lauridsen et al. 2014), ultrasound (Lauridsen et al. 2014), optical coherence tomography (Rival et al. 2015; Tusler et al. 2015; Da Silva et al. 2019) and transmission electron microscopy (Da Silva et al. 2014, 2016) but only one study, on one species of snake, the Carpet python, *Morelia spilotes*, has used scanning electron microscopy to describe the micro‐ornamentation of the surface epithelial cells of the spectacle (Campbell et al. 1999). However, a comprehensive study of nanostructural diversity in the epidermal surface scales in the shed skin of 353 species from 19 families of snakes reveals that the range of nanomorphologies (changes in cell shape and size, cell border type, alterations of the cell surface into holes and channels and the presence of ridges) was predominantly associated with the optical properties, hydrophobicity and friction‐modifying characteristics of the skin rather than habitat type (Hazel et al. 1999; Arrigo et al. 2019 and their associated database https://snake-nanogratings.lanevol.org). Gower (2003), in his study of 20 species of fossorial snakes, came to a similar conclusion, where he considered that friction reduction, dirt shedding and wettability are the main drivers of micro‐ornamentation rather than the control of iridescence given that many features of the Oberhäutchen are smaller than the wavelengths of visible light. The microstructure, including the recesses between scales in the skin of the pelagic yellow‐bellied sea snake, *Hydrophis platurus*, appears to maintain the skin wettability when the dorsal body protrudes above water while floating on calm oceanic slicks where they forage (Lillywhite and Menon 2019). Leaf‐like microstructures striated with nanoridges in the skin, in association with pigmentation, are shown to be responsible for optical changes in texture and improvements in camouflage in the West African Gaboon viper, *Bitis rhinoceros* (Spinner et al. 2013). Changes in pigmentation and tightly interlocking cell junctions, in otherwise smooth, epithelial cells of the skin covering the caudal lure of the Southern death adder, *Acanthophis antarcticus* also imparts a textual change (increase in glossiness) during ontogeny that may affect their ability to entice prey (Crowe‐Riddell et al. 2021).

There has also been little attention given to examining the density of epithelial cells on the surface of the spectacle (or cornea in the absence of a spectacle) in reptiles. In the species examined, spectacular cell densities range from 2283 to 5095 cells/mm^2^ in the Crocodile, *Crocodilis porosus* and the Ornate lizard, *Ctenophorus ornatus*, respectively (Collin and Collin 2000a; Collin and Collin 2006). Similarly, centro‐peripheral changes in the size, shape and density of surface cells in the snake spectacle have been restricted to a single species of Carpet python, *M. spilotes* (Campbell et al. 1999). There is also a paucity of studies on the ultrastructure of the surface cells in the spectacle of snakes, that is, the architectural arrangement of the epithelial cell arrays (cell to cell borders) and the micro‐ornamentation (indentations, pits, pores and microridges). Many authors have described these in the scales of the skin of snakes (Hazel et al. 1999; Campbell et al. 1999; Arnold 2002; Gower 2003; Abdel‐Aal et al. 2010; Spinner et al. 2013, 2014; Arrigo et al. 2019; Toro et al. 2021) and have found species‐specific variation in their size, spacing and depth/height (Price and Kelly 1989) but whether these features are reflected in the surface layers of the spectacle, in light of its ocular role, is largely unknown.

Micro‐ornamentation encompasses a diversity of nanostructures. Micropits over the surface of snake scales are thought to act as spectral filters to reduce reflections of visible, short wavelength light, while still allowing longer wavelength (infrared) parts of the spectrum to be absorbed and used to detect the presence of food and predators (Amemiya et al. 1995; Campbell et al. 1999). Other functions proposed for pores and pits include increasing the ability to shed dirt from the skin surface (Gower 2003) and repel water (Arrigo et al. 2019). Microridges also adorn the surface of the skin in some species of snakes with a diversity of patterns (Arrigo et al. 2019), with similarities to those found over the cornea of other (non‐spectacled) species of reptiles (Collin and Collin 2000b). These microridges are comprised of a combination of intermediate, actin and keratin filaments to maintain structural integrity (Uehara et al. 1991) and have a range of proposed functions including contributing to hydrophobicity and dirt shedding (Gower 2003; Spinner et al. 2014; Arrigo et al. 2019), reducing reflection as an anti‐predator strategy (Arnold 2002) and facilitating the spread of mucus to maintain hydration and stabilise the corneal tear film (Sperry and Wassersug 1976; Hodges and Dartt 2013). However, a number of species of snakes are devoid of surface ornamentation (holes, micropits or microridges), where the surface of the epithelial cells appears to be completely smooth and the Oberhäutchen unperforated (Gower 2003; Arrigo et al. (2019).

The aims of this morphological study are to examine the topographic changes in density, size, shape, interdigitation and micro‐ornamentation of epithelial cells on the surface of the spectacle in the eyes of a range of land (four species) and sea (five species) snakes using a variety of imaging techniques. The results reveal species‐specific differences that may reflect functional adaptations and improve our understanding of the role of the “spectacle scale” in snake vision, compared to extraocular (head and body) scales.

## 2 Methods

### 2.1 Source of Animals

Nine species of snakes (four aquatic/marine, one semiaquatic marine and four terrestrial) were used in this study (Table 1) predominately from the front‐fanged venomous (Elapidae) snakes from the subfamily Hydrophinae, and a single pit viper species from the family Viperidae. Up to three individuals per species (six spectacles) were used but in some cases only a single individual was captured providing only two spectacles. The cornea, eyes, other sense organs and brains of these animals were all used for various other studies. Adult snakes were collected by dipnet at the surface between 1 and 10 km off the coast of Exmouth, Broome and/or off the Kimberley coast in Western Australia, Australia (sea snakes) or sourced from a commercial provider (Venom Supplies Pty Ltd, Tanunda, South Australia) between May 2022 and July 2024 with approval of The University of Adelaide Animal Ethics Committee (Science) (S‐2021‐017), the Western Australian Government (collection permit number FO25000393) and the Western Australian Department of Biodiversity, Conservation and Attractions (SF010002). All interactions with captive animals were conducted under the requirements of the Department for Environment and Water and the institutional guidelines of Venom Supplies, and were undertaken in conformance with the Animal Welfare Act 1985 (South Australia). All handling of snakes required personal protective equipment (PPE) given the venomous nature of these reptiles and all translocation, monitoring and euthanasia followed strict guidelines (Chapman et al. 2008).

**Table 1:** Information about the nine study species including their ecology, capture location, habitat and individual morphometric data.

Ecology	Specimen code	Common name (Family)	Genus species	Capture location	Sex	Mass (g)	SVL (cm)	Tail length (cm)	Total length (cm)	Head Length (cm)	Head width (cm)	Fixation	Habitat
Aquatic/marine	KLS1382	Elegant sea snake (Elapidae)	*Hydrophis elegans* [Gray 1842]	Kimberley, Australia	F		107	10	117	NR	NR	Karnovsky's	Coral reefs and shallow, sandy or muddy bottom
Aquatic/marine	KLS1631	Stoke's sea snake (Elapidae)	*Hydrophis stokesii* [Gray 1846]	Kimberley, Australia	M	445	78	13	NR	NR	NR	Karnovsky's	Found in structured habitats such rocky/coral bommies and sponge gardens
Aquatic/marine	KLS1691	Stoke's sea snake (Elapidae)	*Hydrophis stokesii* [Gray 1846]	Broome, Australia	F	835	108	15	NR	NR	NR	Karnovsky's	Found in structured habitats such rocky/coral bommies and sponge gardens
Aquatic/marine	KLS1656	Elegant sea snake (Elapidae)	*Hydrophis elegans* [Gray 1842]	Broome, Australia	M	345	115	11	NR	NR	NR	Karnovsky's	Coral reefs and shallow, sandy or muddy bottom
Aquatic/marine	KLS1453	Olive sea snake (Elapidae)	*Aipysurus laevis* [Lacépède 1804]	Exmouth, Australia	F	1300	114	16	130	NR	NR	Karnovsky's	Found in structured habitats such rocky/coral bommies and sponge gardens
Aquatic/marine	KLS1623	Olive sea snake (Elapidae)	*Aipysurus laevis* [Lacépède 1804]	Broome, Australia	M	NR	NR	NR	NR	NR	NR	Karnovsky's	Found in structured habitats such rocky/coral bommies and sponge gardens
Aquatic/marine	KLS1383	Bar‐bellied sea snake (Elapidae)	*Hydrophis major* [Shaw 1802]	Kimberley, Australia	M	NR	81	12	93	NR	NR	4% PFA	Range of habitats including coral reefs and seagrass beds, lagoons
Aquatic/marine	KLS1456	Bar‐bellied sea snake (Elapidae)	*Hydrophis major* [Shaw 1802]	Exmouth, Australia	F	NR	118	17	135	NR	NR	Karnovsky's	Range of habitats including coral reefs and seagrass beds, lagoons
Aquatic/marine	KLS1502	Bar‐bellied sea snake (Elapidae)	*Hydrophis major* [Shaw 1802]	Exmouth, Australia	M	1150	117	32	149	NR	NR	Karnovsky's	Range of habitats including coral reefs and seagrass beds, lagoons
Semi Aquatic/marine	KLS1659	Black‐ringed sea snake (Elapidae)	*Hydrelaps darwiniensis* [Boulenger 1896]	Broome, Western Australia	M	< 50	26	3.5	29.5	NR	NR	Karnovsky's	Mangroves and on sandy flats. Shallow depths
Terrestrial	JCR0049	Eastern tiger snake (Elapidae)	*Notechis scutatus* [Peters 1861]	Kangaroo Island, South Australia	F	1069	137.5	21.5	159	NR	NR	4% PFA	Ground‐dwelling but are able to swim and climb into trees
Terrestrial	MJF0062	Cantil viper (Viperidae)	*Agkistrodon bilineatus* [Günther 1863]	Venom Supplies, South Australia	F	566	87.5	17.8	NR	45	40	4% PFA	Thick vegetation and forests
Terrestrial	MJF0063	Cantil viper (Viperidae)	*Agkistrodon billineauts* [Günther 1863]	Venom Supplies, South Australia	M	490	70.0	19.4	NR	44	47	4% PFA	Thick vegetation and forests
Terrestrial	MJF0060	Black tiger snake (Elapidae)	*Notechis ater* [Cogger 1983]	Venom Supplies, South Australia	M	616	88.0	12.1	NR	33	33	4% PFA	Ground‐dwelling but are able to swim and climb into trees
Terrestrial	MJF0061	Black tiger snake (Elapidae)	*Notechis ater* [Cogger 1983]	Venom Supplies, South Australia	M	808	105.0	18.4	NR	30	30	4% PFA	Ground‐dwelling but are able to swim and climb into trees
Terrestrial	MJF0065	Black tiger snake (Elapidae)	*Notechis ater* [Cogger 1983]	Venom Supplies, South Australia	F	919	107.0	15.6	NR	40	43	4% PFA	Ground‐dwelling but are able to swim and climb into trees
Terrestrial	JCR0039	Coastal taipan (Elapidae)	*Oxyuranus scutellatus* [Peters 1867]	Captive bred at Venom Supplies, South Australia	F	1360	166.8	28.5	NR	NR	NR	4% PFA	Monsoon forests to open woodland and sugarcane fields
Terrestrial	NR	Coastal taipan (Elapidae)	*Oxyuranus scutellatus* [Peters 1867]	Venom Supplies, South Australia	NR	NR	NR	NR	NR	NR	NR	4% PFA	Monsoon forests to open woodland and sugarcane fields

All snakes were euthanised with a lethal injection into the body cavity of diluted pentobarbitone (6 mg/mL) at a dosage of 150 mg/kg using a 23–30 G needle in accordance with the guidelines of the Australian Code of Practice for the Care and Use of Animals for Scientific Purposes, under Animal Ethics Committee protocols from The University of Adelaide (S/2021‐017) and The University of Western Australia (RA/3/100/1369). The heads (including the eyes and ocular spectacle of all snake specimens were fixed by immersion in either 4% paraformaldehyde in 0.1 mol L^−1^ phosphate buffer or Karnovsky's solution (2% paraformaldehyde and 2.5% glutaraldehyde in 0.1 mol L^−1^ phosphate buffer) for histology and light microscopy (using paraffin embedding) and either scanning or transmission electron microscopy. The surface of the spectacle and surrounding scales of selected species were also examined using gel‐based stereo profilometry. Determination of sex and measurement of mass, snout to vent length, tail length and total length were recorded (where possible) using digital callipers or a tape measure (Table 1) before preservation. It was not possible to ascertain the stage of ecdysis, which we presume could have been different for each individual and species. Therefore, the influence of this process on the number and distribution of any micro‐ornamentation (micropits, microholes or microridges) is unknown.

**Figure 1:**
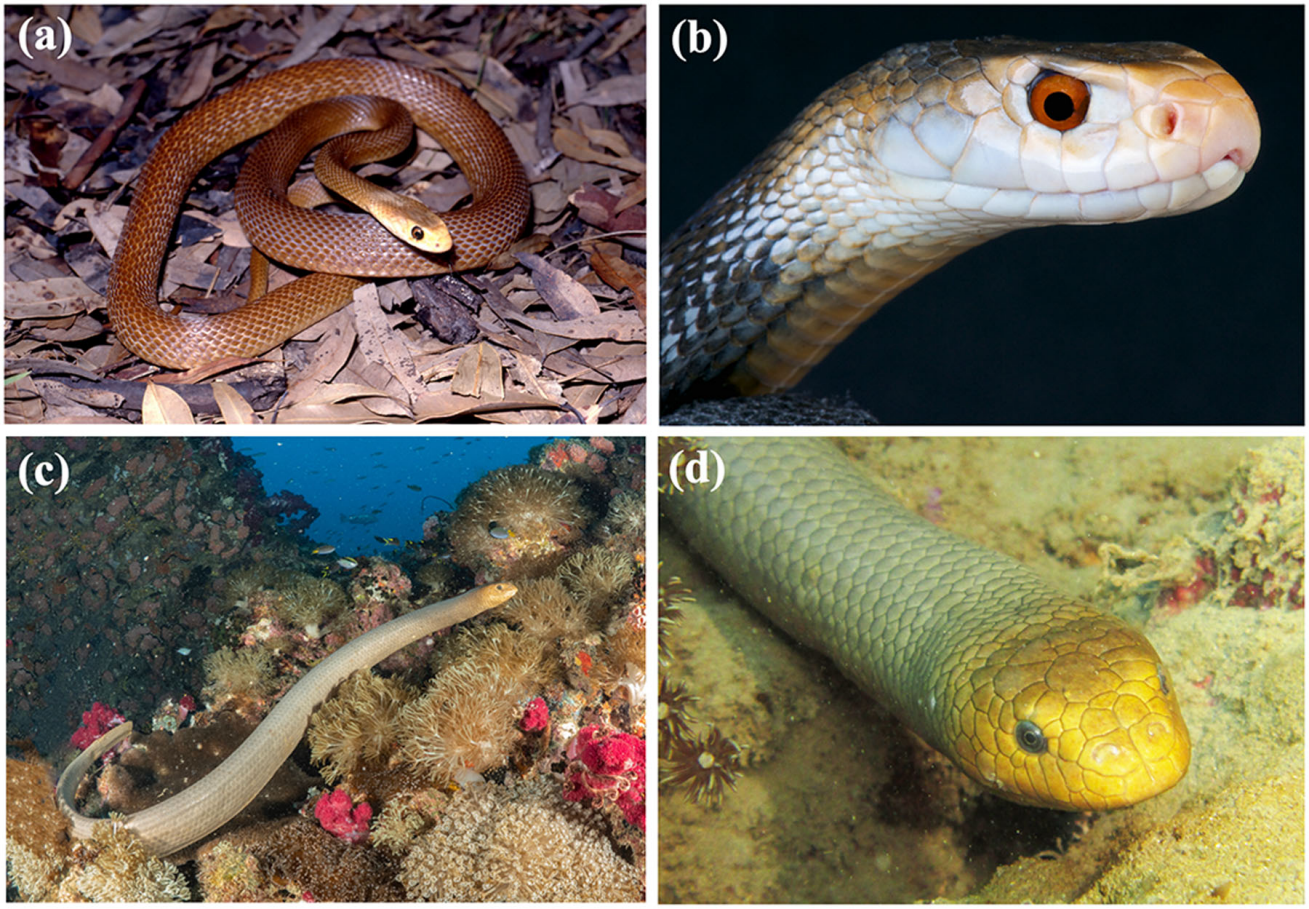
(a) Photograph of the coastal taipan *Oxyuranus scutellatus* sitting on leaf litter. (b) Close up of the head of *O. scutellatus* showing the prominent eyes (including red iris) surrounded by head scales. (c) Photograph of the olive brown sea snake *Aipysurus laevis* within its natural coral reef habitat. (d) Close up of *A. laevis* showing its dark iris and relatively small eyes.

After immersion fixation for 24 h, the eyes, including the overlying ocular spectacle and surrounding scale tissue, were carefully dissected free of the orbits within the head. The spectacle was carefully separated from the underlying cornea using a continuous radial incision in the conjunctival region of the eye at the level of the circumocular sulcus, although for some individuals extraocular tissue including parts of the (head) scales were also removed (Figure 1). Whole spectacles and extraocular scales were washed and stored in 0.1 mol L^−1^ phosphate buffer (pH 7.4) with 0.05% sodium azide at 4°C until processing and analysis.

### 2.2 Micro Computed Tomography

A single head of *Hyrophis major* from the South Australian Museum (SAMA R67292) that had previously been fixed in 10% formalin and stored in 70% ethanol was prepared for soft tissue micro computed tomography (µCT) scanning. The head was rehydrated over two to 3 days using a series of decreasing ethanol solutions (70%, 50%, 25%) before being immersed in 1.25% buffered Lugol's solution (I_2_ + KI + H_2_O + Na_2_HPO_4_ + KH_2_PO_4_) following Dawood et al. (2021). The head was µCT scanned using a Phoenix Nantom (Waygate Technologies, PA, USA) at The Melbourne Trace Analysis for Chemical, Earth and Environmental Sciences. The sample was scanned with a 0.5 mm aluminium filter for 15 min with an X‐ray tube voltage of 120 kV and a current of 300 µA collecting 1199 2D x‐ray projections from different angles through a full 360‐degree rotation of the sample. Tomographic projections were reconstructed and cross‐sectional images obtained using Avizo3D Pro version 2022.3 (ThermoFisher Scientific, MA, USA).

### 2.3 Histology and Light Microscopy

The eyes (with intact spectacle) of selected species were prepared for paraffin embedding using a tissue processor (Leica ASP300S). Tissue was moved through a series of dehydration steps (70%, 90%, 3 × 100% EtOH and 3x xylene at 37°C) before gradually being infiltrated with paraffin wax at 60°C. Infiltrated samples were then manually embedded in paraffin wax on an embedding station (Leica Histocore Arcardia H) in a transverse orientation. Freshly embedded blocks were left to set on a cooling plate at −4°C (Leica Histocore Arcardia H) for 20 min, then stored in sealed plastic containers. The paraffin blocks were then sectioned using a semi‐automatic microtome (Leica RM2135) at 10 µm, collected and mounted on poly‐lysine coated microscope slides (Thermo Fischer Scientific), and then cured for 2 h in the oven at 60°C. All slides were stained with solutions of Harris haematoxylin (Harris haematoxylin mercury free, POCD Scientific, VWRC351945S) and 0.25% ethanol‐based eosin (Eosin‐Y Sigma‐Aldrich, 230251). Stained paraffin sections were then coverslipped and examined using a BH‐2 Olympus compound light microscope and photographed on an Olympus DP‐30 digital camera fitted with a trinocular C mount.

The whole eye, anterior segment (including iris, cornea and spectacle) and/or only the spectacle was also examined in transverse section using light microscopy in resin making sure to include both central and peripheral regions. For this analysis, tissue was post‐fixed in 1% (w/v) osmium tetroxide (Sigma‐Aldrich) in 0.13 mol L^−1^ Sorenson's buffer (0.13 mol L^−1^ Na_2_HPO_4_, Millipore Sigma, 0.13 mol L^−1^ KH_2_PO_4_, Sigma‐Aldrich, pH 7.4) for about 2 h. The tissue was rinsed in 0.13 mol L^−1^ Sorenson's buffer, dehydrated through a graded series of alcohols (25%, 50%, 70%, 80%, 95% and 100% EtOH) followed by immersion in propylene oxide (VWR), before infiltration with araldite (ProSciTech). Resin capsules were then cured at 60°C overnight. Transverse (semithin) Section (1–2 μm) were cut using an American Optical rotary microtome and either a glass or diamond knife (DiATOME 45°). Resin sections were mounted on subbed slides, stained with Toluidine blue, examined using a BH‐2 Olympus compound light microscope and photographed on an Olympus DP‐30 digital camera fitted with a trinocular C mount.

### 2.4 Scanning Electron Microscopy (SEM)

At least one spectacle (but typically two to three if available) and an adjacent scale from different individuals were processed for SEM to examine the surface micro‐ornamentation and the topography of epithelial cells. Preserved tissues were rinsed in three changes of 0.1 mol L^−1^ Sorensen's buffer, each for 10 min and post‐fixed in 1% osmium tetroxide (OsO_4_) in 0.1 M Sorensen's buffer for 1 h. The spectacle and scales were then rinsed in two changes of milliQ water, each for 15 min, and dehydrated in a series of alcohols (30%, 50%, 70%, 80%, 90%, 95% and 100% EtOH), each for 20 min, and critical point dried for 1.5 h using a Quorum E3000 Critical Point Dryer (Quorum Technologies Ltd., Laughton, East Sussex, UK). Samples were sputter coated with a Polaron SC7640 Sputter Coater with a platinum target (Polaron, Watford, UK) for approximately 90 s. The samples were imaged using an Hitachi Ultrahigh‐Resolution Schottky Scanning Electron Microscope (model SU7000, Hitachi, Tokyo, Japan) equipped with backscattering secondary electron and STEM detectors in high vacuum mode with accelerating voltages of between 3 and 7 kV, and a spot size of 30. Each spectacle and scale was hemisected so that half of the tissue was inverted and both sides were displayed to ensure both anterior and posterior surfaces were differentiated. Each piece of tissue was then mounted on a 10 mm aluminium stub with double‐sided tape, making sure of the orientation. Digital images were produced using the lower, upper and middle detectors. Navigation images of each of the spectacles or scales were taken by stitching together low magnification images of the surface of each spectacle/scale. SEM images were measured using the Fiji open‐source platform for biological‐image analysis (Schindelin et al. 2012), software version 1.8.0_332 (64‐bit Java, Image J 1.54).

### 2.5 Gel‐Based Profilometry

A GelSight 1.0X Mobile Core (GelSight Inc., Waltham, Massachusetts, USA) was used to capture a three dimensional profile of the surface topography of the spectacle of *Hydrophis major* (*n *= 2). Surface topography models were analysed using the GelSight software (version 3.1.156.0). GelSight is a non‐destructive technique that captures high‐resolution surface information from any surface, over an area of 8.508 × 7.099 mm, using gel‐based profilometry. Although not able to capture micro‐ornamentation, the Gelsight provided information of the size, shape, curvature and relative position of the spectacle relative to the surrounding scales (Wagner et al. 2025).

### 2.6 Quantitative Analyses

The quantitative analyses of the surface cell topography and micro‐ornamentation were primarily undertaken using digital images generated using SEM. Cell area (µm^2^), cell density (cells/mm^2^), cell shape, cell length (µm), hole diameter (µm) and microridge thickness (µm) (where present) and the tortuosity of the epithelial cell borders were all measured across a centro‐peripheral transect (a line from the centre to the periphery) of the spectacle of each species. For three species (the Coastal taipan *Oxyuranus scutellatus*, the Eastern tiger snake *Notechis* scutatus and the Elegant sea snake *Hydrophis elegans)*, the spectacle was (digitally) divided into six regions (200–250 µm in diameter) to investigate any subtle ultrastructural changes in surface ornamentation from centre to periphery in two terrestrial and one aquatic species. Cell density (cells/mm^2^), hole diameter (µm), microridge thickness (µm) and cell length (µm) were also measured topographically in all species, showing differences between central (transectoral range of 800–1200 µm) and peripheral (transectoral range of 1–500 µm) regions of the spectacle. Measurements were obtained by digital analysis of the computer images using NIH Image. The sample number for all surface features measured for each species varied (*n* = 25–230) but provided a robust mean and standard deviation (±SD).

Quantitative data were compared using either one‐ or two‐tailed *t*‐tests for independent variables. The central and peripheral regions of the cornea were analysed in all species and both left and right eyes were compared. However, all features were found to be not significantly different for size‐matched individuals (where *n* > 1) and in left and right eyes, as has been found for ocular features in a range of other vertebrate eyes (Nam et al. 2006; Werther et al. 2017; Collin et al. 2021). Therefore, the data for left and right eyes were pooled. No attempt was made to assess the degree of shrinkage in our study to allow direct comparison to be made with measurements derived using previously published scanning or transmission electron micrographs using similar methods. Shrinkage of around 30%–40% is typically expected following fixation and resin embedding for histology and TEM (Hayat 1986). Therefore, a correction factor should be applied to the data presented to give an estimate of the in vivo dimensions of cells and their micro‐ornamentation.

## 3 Results

### 3.1 General Structure of the Snake Spectacle

**Figure 2:**
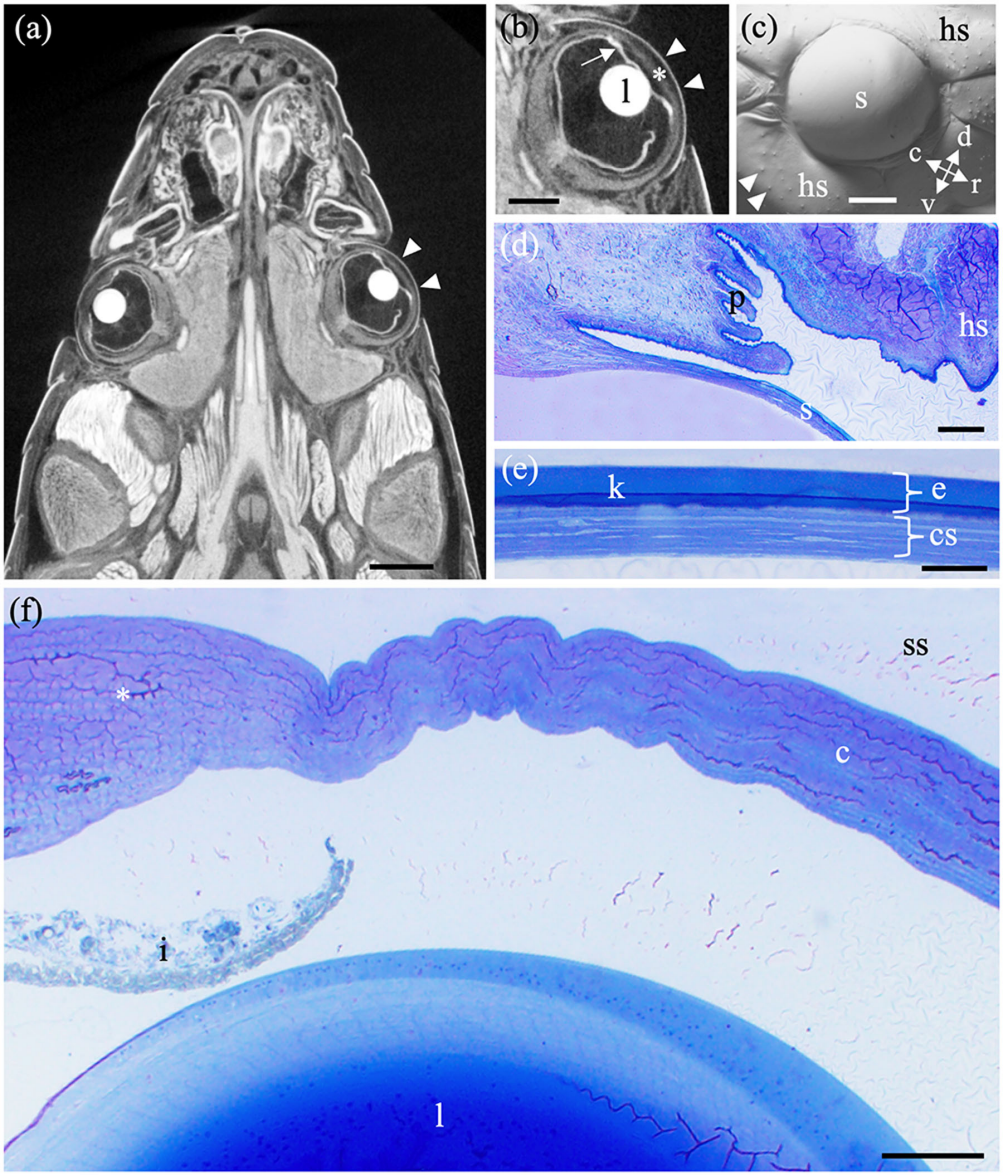
(a) Micro computed tomography generated (µCT) image in the horizontal plane showing sections of the two laterally‐placed eyes within the head of the bar‐bellied seasnake, *Hydrophis major* following contrast enhancement with Lugol's solution. Note the near‐spherical lens of the elliptically shaped eyes. The convex spectacle (arrowheads) covering the eyes invaginates deeply around the circumference of the eyes, before it emerges to cover the head scales. (b) Higher magnification µCT image of the eye of *H. major* in horizontal section showing the outline of the spectacle (arrowheads), the sub‐spectacular space in between the spectacle and the cornea (asterisk) and the iris (arrow). (c) Image of the surface of the ocular spectacle (s) and surrounding head scales (hs) of the bar‐bellied sea snake *H. major* using gel‐based stereo profilometry. Note the convex curvature of the spectacle and its proximity to the adjacent head scales, some of which contain raised mechanoreceptors (arrowheads). Orientation: c, caudal; d, dorsal; r, rostral; v, ventral. (d) Transverse resin section of the spectacle (s) of the Eastern tiger snake *Notechis scutatus* showing it invaginates in the periphery of the eye to form several finger‐like processes (p), which transition to form the surface (keratinised layer) of a head scale (hs). (e) High magnification of a transverse section of the spectacle in *N. scutatus* stained with Toluidine blue showing the outer (keratinised) layer (k) within a zone of epithelial cells (e) and a stroma containing collagen lamellae (cs). (f) Transverse section of the anterior segment, comprising the cornea (c), iris (i) and lens (l) in the lateral region of the eye in *N. scutatus* showing the thickening of the cornea peripherally (asterisk). The spectacle and the underlying cornea are separated by a sub‐spectacular space (ss). Scale bars 2 mm (a); 1 mm (b, c); 200 µm (d); 50 µm (e); 200 µm (f).

The eyes of both the land and sea snakes examined are all laterally‐placed and surrounded by a mosaic of head scales (Figures 1 and 2a,b). Eye size, iris colour, and the degree to which the eyes protrude beyond the contour of the head varied significantly between species (Figure 1). The spectacles in all study species are composed of collagen fibres bounded anteriorly by an epithelium and posteriorly by an endothelium. The spectacle is separated from the cornea by a sub‐spectacular space. The curvature of the spectacle follows closely that of the eye and underlying cornea but there are significant inter‐specific differences in the degree of convexity, the adherence of the spectacle to the cornea and the depth to which the spectacle invaginates around the periphery of the eyecup before it transitions into the surrounding head scales (Figure 2c,d). The outer surface of the epithelium in both the spectacle and the scales progressively becomes keratinised to form the Oberhäutchen (Arrigo et al. 2019; Gower 2003), which is shed when the snake undergoes periodic ecdysis. The Oberhäutchen over the spectacle and head scales (in addition to the iris and the lens) appear as highly contrasted (bright) surfaces following micro computed tomography scans (Figure 2a,b). The thickness of the spectacle does not change significantly from centre to periphery, in contrast to the cornea (Figure 2d–f).

### 3.2 Topographic Changes in the Density, Shape and Size of Spectacular Surface Cells

**Figure 3:**
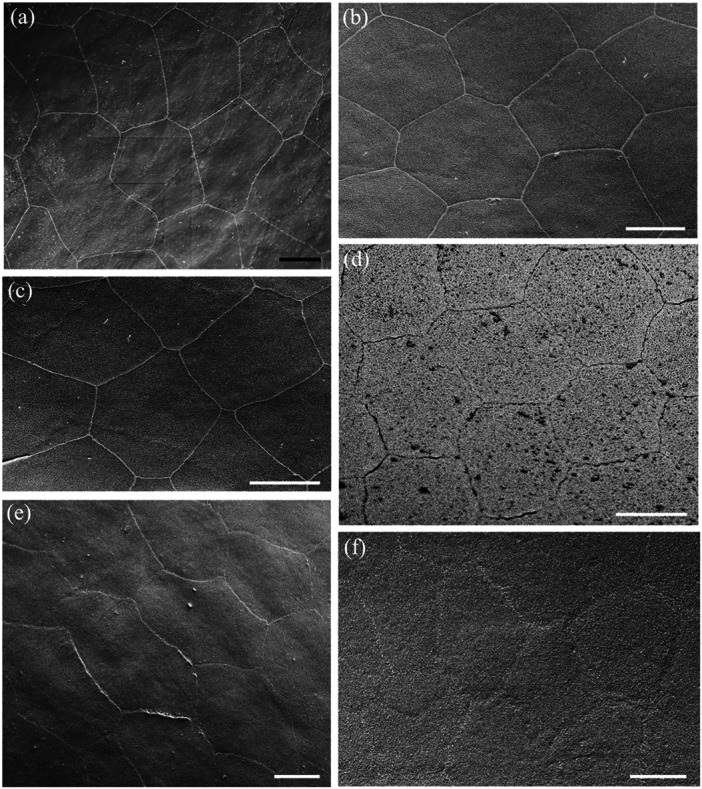
Scanning electron micrographs of the surface of the epithelial cells of the central spectacle showing their pentagonal or hexagonal shapes and straight cell borders in both land and sea snakes. (a) *Hydrophis major*. (b and c) *Hydrelaps darwiniensis*. (d) *Oxyuranus scutellatus*. (e) *Hydrophis stokesii*. (f) *Notechis ater*. Scale bars 10 µm (a–f).

**Figure 4:**
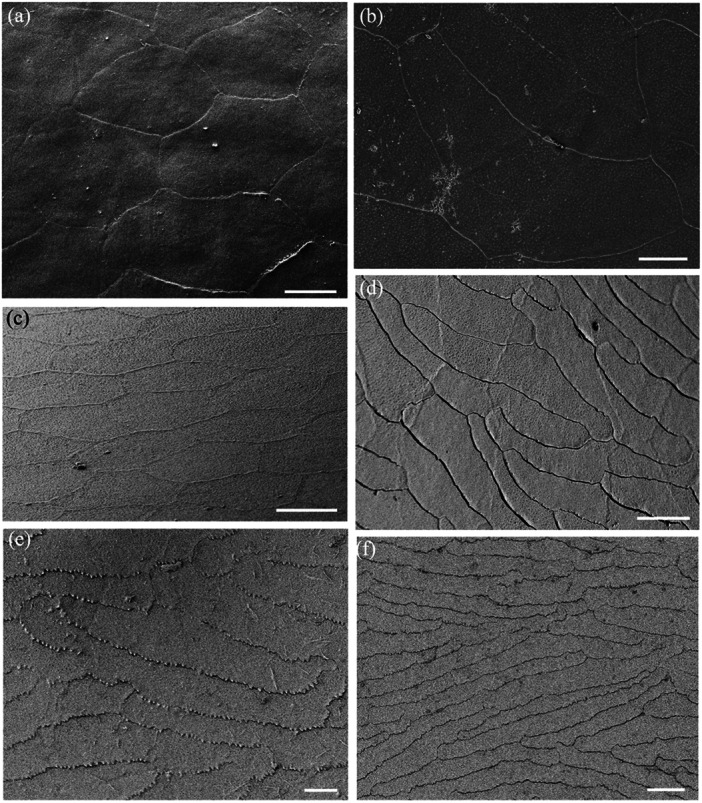
Scanning electron micrographs of the surface of the epithelial cells of the transitional zone between the centre and periphery of the spectacle showing the variations in cell shape and size. Note the elongation in cell shape. (a) *Hydrophis stokesii*. (b) *Aipysurus laevis*. (c) *Notechis scutatus*. (d–f) *Notechis ater*. The periphery of the spectacle is towards the bottom or bottom left corner of each micrograph. Scale bars 10 µm (a); 5 µm (b); 10 µm (c, d); 5 µm (e); 10 µm (f).

**Figure 5:**
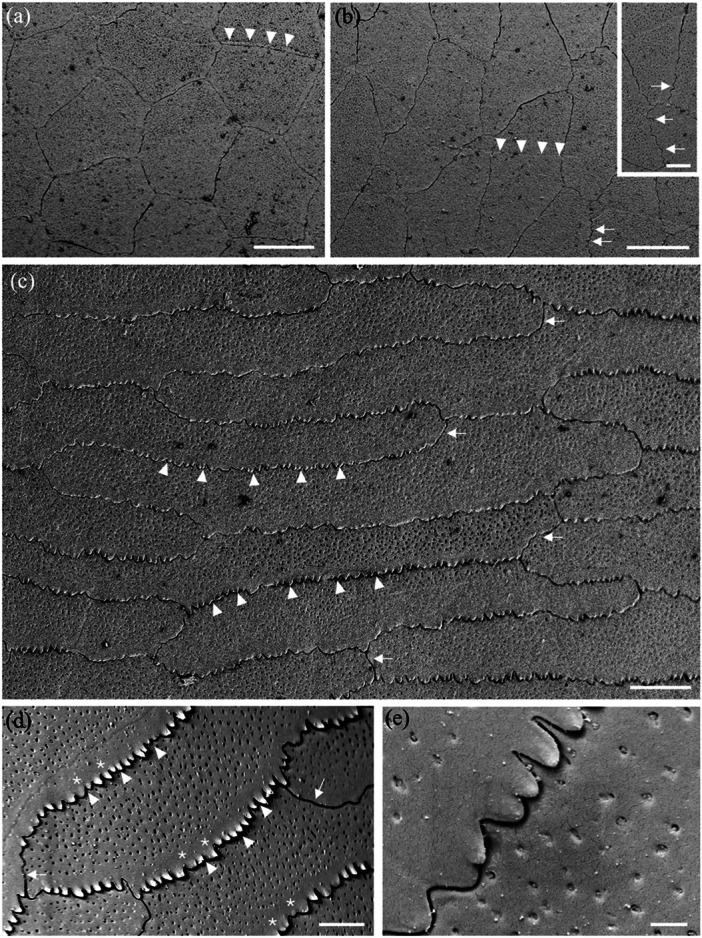
Scanning electron micrographs of the surface of the epithelial cells of the spectacle in *Oxyuranus scutellatus* showing the changes in cell size, shape and cell borders from centre to periphery. (a) Cells within the centre of the spectacle showing the repeating pattern of pentagonal epithelial cells. Row of arrowheads depicts the surface impression of previously shed epithelial cell boundaries. (b) Cells within the intermediate zone showing the cells have become elongated with irregular cell borders, which are highlighted in the inset (arrows). (c) Very long cells within the periphery of the spectacle showing the sawtooth (deep zigzag) borders between cells (arrowheads) along the cell border that is parallel to the circumferential (peripheral) edge of the spectacle. Note these zigzag borders give way to smooth, irregular borders at each end of the cell (arrows). (d) Close up of the two types of cell borders: deep zigzag (arrowheads) and smooth (arrows). Note the absence of microholes or micropits along the peripheral border of each cell (asterisks). (e) Deep zigzag interdigitations between adjacent epithelial cells in the periphery of the spectacle. Note that the tips of each saw are slightly raised. Scale bars 10 µm (a); 10 µm (b); 3 µm (b inset); 5 µm (c); 2 µm (d); 0.5 µm (e).

**Figure 6:**
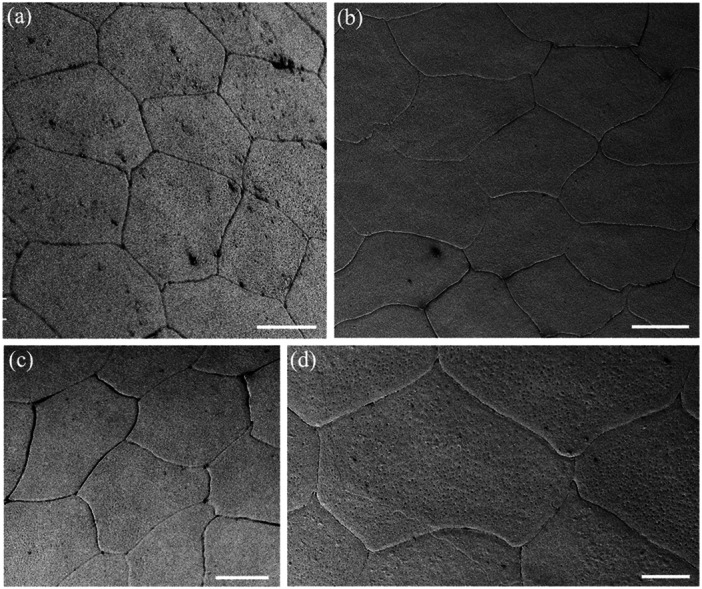
Scanning electron micrographs of the surface of the epithelial cells of the spectacle in *Hydrophis elegans* showing the changes in cell size and shape from centre to periphery. (a) Cell array within the centre of the spectacle showing pentagonal and hexagonal cells with straight cell borders. (b and c) Transitional zone between the centre and periphery showing the elongation of some cells. (d) Larger and slightly elongated epithelial cells in the periphery, some which form curved borders. Scale bars 10 µm (a–c); 5 µm (d).

The spectacle of all the snake species examined is covered by a layer of keratinised epithelial cells with cell densities ranging from 2579 ± 752 cells/mm^2^ to 10,391 ± 850 cells/mm^2^ in the centre and from 2315 ± 532 cells/mm^2^ to 4291 ± 771 cells/mm^2^ in the periphery (Table 2, Figures 3, 4, 5, 6). One semi‐aquatic (*Hydrelaps darwiniensis*) and one terrestrial (*Agkistrodon bilineatus*) species have similar densities in both the centre and periphery, while the remaining seven species have significantly decreased peripheral densities.

**Table 2:** Differences in the density of epithelial cells at the surface of the spectacle of the nine study species from central to peripheral regions analysed from digital images obtained using scanning electron microscopy. The percentage differences and statistical significance are also provided.

Common name	Species	Central cell density	*n*	Peripheral cell density	*n*	% difference	Significance
Terrestrial							
Black tiger snake	*Notechis ater*	4148 ± 622 cells/mm^2^	75	3575 ± 1053 cells/mm^2^	62	−16.0	*p* = 0.00036
Coastal taipan	*Oxyuranus scutellatus*	5501 ± 1123 cells/mm^2^	87	4291 ± 771 cells/mm^2^	91	–21.5	*p* < 0.00001
Eastern tiger snake	*Notechis scutatus*	10,391 ± 850 cells/mm^2^	79	3558 ± 412 cells/mm^2^	106	–192	*p* < 0.00001
Mexican moccasin	*Agkistrodon bilineatus*	2579 ± 752 cells/mm^2^	36	2772 ± 343 cells/mm^2^	76	+7.5	*p* = 0.075
Aquatic							
Bar‐bellied sea snake	*Hydrophis major*	2905 ± 867 cells/mm^2^	68	2552 ± 817 cells/mm^2^	81	–8.8	*p* = 0.015
Black‐ringed sea snake	*Hydrelaps darwiniensis*	3866 ± 475 cells/mm^2^	84	3906 ± 546 cells/mm^2^	84	+1.0	*p* = 0.629
Elegant sea snake	*Hydrophis elegans*	4485 ± 2571 cells/mm^2^	105	2315 ± 532 cells/mm^2^	118	–93.7	*p* < 0.00001
Olive‐brown sea snake	*Aipysurus laevis*	3003 ± 469 cells/mm^2^	44	2478 ± 298 cells/mm^2^	35	–21.2	*p* < 0.00001
Stoke's sea snake	*Hydrophis stokesii*	3146 ± 588 cells/mm^2^	75	2309 ± 461 cells/mm^2^	95	–36.2	*p* < 0.00001

The cells in the centre of the spectacle of all nine species have similar shapes, being mostly hexagonal or pentagonal (Figure 3). Most have straight sides, although the cell margins of the Eastern tiger snake, *Notechis scutatus* and the Cantil viper, *A. bilineatus* show marked interdigitations (Figure 4c). In the periphery, all species show an increase in the length of their surface spectacular cells ranging from an increase of 5.4% in *A. bilineatus* to 165% in the Coastal taipan, *Oxyuranus scutellatus* (Tables 3, 4, 5, 6). The greatest increases across the nine species are in the *O. scutellatus* (165%), the Black tiger snake, *Notechis ater* (148%) and the Eastern tiger snake, *Notechis scutatus* (94.6%), in all of which the cells are oriented circumferentially (Table 6). The majority of these peripheral cells have straight sides, i.e. without interdigitations, although *N. ater* and *O. scutellatus* have very marked interdigitations (zigzags) and *A. bilineatus* has irregular cell margins (Figure 5). In those species with very marked interdigitations in the peripheral cells (*N. ater* and *O. scutellatus*), the ends of the long cells show no interdigitations (Figure 5).

**Table 3:** Quantitative assessment of the changes in superficial epithelial cells morphology in the spectacle of the Coastal taipan, *Oxyuranus scutellatus* from the periphery to the centre within six zones. Cell area, density, shape, length and border type and hole diameter are all examined across these transectional regions.

Zone	1		2		3		4		5		6	
Distance from periphery (µm)	1−250	*n*	250−500	*n*	500−700	*n*	700−900	*n*	900‐1000	*n*	1000–1200	*n*
Cell area—Mean (µm^2^)	257.9 ± 27.1	74	234.3 ± 24.3	48	223.9 ± 14.9	35	209.2 ± 31.3	36	170.9 ± 19.6	48	129.4 ± 15.6	36
Cell area—Range (µm^2^)	206.6−303.3	74	198.1−345.0	48	191.7−252.7	35	129.0−270.3	36	140.3–222.3	48	98.1–156.7	36
Cell density (cells/mm^2^)	3877.5 ± 407.4	74	4268.0 ± 442.6	48	4466.2 ± 297.2	35	4780.1 ± 715.2	36	5851.4 ± 671.1	48	7728.0 ± 931.7	36
Cell shape	Very long		Long		Elongated		Oval		Rounded polygonal		Polygonal	
Cell length—Mean (µm)	51.0 ± 5.9	51	44.8 ± 5.8	43	33.1 ± 3.5	48	22.5 ± 3.6	59	18.9 ± 1.7	41	18.6 ± 2.2	39
Cell length—Range (µm)	43.2−67.2	51	33.3−61.7	43	25.7−38.4	48	17.1−30.8	59	16.2–22.5	41	12.0–23.1	39
Hole diameter—Mean (nm)	132.5 ± 36.5	58	112.1 ± 24.1	42	101.3 ± 30.3	82	100.1 ± 29.3	45	81.1 ± 118.0	44	59.5 ± 17.1	43
Hole diameter—Range (nm)	68.0−220.7	58	53.4−160.5	42	42.7−160.2	82	53.4−160.1	45	51.1–128.1	44	40.4–106.6	43
Cell borders	Deep zigzag		Shallow zigzag		Irregular		Irregular		Curved		Straight	

**Table 4:** Quantitative assessment of the changes in superficial epithelial cells in the spectacle of the Eastern tiger snake, *Notechis scutatus* from the periphery to the centre within six zones. Cell area, density, shape, length and border type and hole diameter are all examined across these transectional regions.

Zone	1		2		3		4		5		6	
Distance from periphery (µm)	1–250	*n*	250–500	*n*	500–700	*n*	700–900	*n*	900–1000	*n*	1000–1200	*n*
Cell area—Mean (µm^2^)	295.8 ± 28.2	62	259.4 ± 26.1	43	203.1 ± 25.6	38	193.0 ± 19.4	28	96.0 ± 8.1	38	96.1 ± 7.8	39
Cell area—Range (µm^2^)	210.1–358.0	62	193.6–298.8	43	134.6–242.3	38	159.3–233.2	28	78.3–110.9	38	78.5–110.6	39
Cell density (cells/mm^2^)	3390.7 ± 323.3	62	3855.1 ± 387.9	43	4923.7 ± 620.6	38	5181.3 ± 520.8	28	10,416.7 ± 878.9	38	10,405.8 ± 844.5	39
Cell shape	Very long		Long		Elongated		Oval		Rounded polygonal		Polygonal	
Cell length—Mean (µm)	50.0 ± 6.1	31	41.7 ± 4.9	36	35.1 ± 5.9	27	24.4 ± 1.7	53	25.2 ± 2.7	31	21.6 ± 1.9	31
Cell length—Range (µm)	42.7–65.1	31	29.2–49.8	36	25.2–44.1	27	24.2–28.1	53	18.8–28.8	31	16.6–24.6	31
Hole diameter—Mean (nm)	134.0 ± 30.1	36	107.5 ± 23.2	36	87.0 ± 25.2	52	67.3 ± 20.6	41	63.5 ± 21.9	51	55.2 ± 16.4	62
Hole diameter—Range (nm)	76.6–178.7	36	64.1–170.2	36	42.4–133.8	52	38.3–153.1	41	25.5–110.6	51	25.6–85.5	62
Cell borders	Slightly curved		Slightly curved		Irregular		Irregular		Curved		Straight	

**Table 5:** Quantitative assessment of the changes in superficial epithelial cells in the spectacle of the Elegant seasnake *Hydrophis elegans* from the periphery to the centre within six zones. Cell area, density, shape, length and border type and hole diameter are all examined across these transectional regions.

Zone	1		2		3		4		5		6	
Distance from periphery (µm)	1–200	*n*	200–400	*n*	400–600	*n*	600–800	*n*	800–900	*n*	900–1000	*n*
Cell area—Mean (µm^2^)	473.3 ± 92.8	63	383.9 ± 85.3	55	362.5 ± 548	51	330.7 ± 45.9	59	258.0 ± 44.9	52	188.6 ± 47.2	53
Cell area—Range (µm^2^)	281.3–680.6	63	241.5–650.5	55	227.3–493.3	51	232.4–433.9	59	159.75–354.3	52	86.3–280.6	53
Cell density (cells/mm^2^)	2112.8 ± 412.6	63	2605.0 ± 379.0	55	2758.5 ± 575.7	51	3024.2 ± 420.1	59	3,875.7 ± 674 ± 38	52	6,002.4 ± 1700.2	53
Cell shape	Elongated		Elongated		Rounded polygonal		Rounded polygonal		Polygonal		Polygonal	
Cell length—Mean (µm)	33.7 ± 5.3	58	32.0 ± 4.8	37	28.3 ± 3.4	41	27.2 ± 3.0	35	24.1 ± 2.6	39	21.0 ± 3.4	34
Cell length—Range (µm)	22.3–48.2	58	23.0–41.8	37	21.8–35.3	41	22.5–36.7	25	19.74 ± 32.8	39	14.1–26.6	34
Hole diameter—Mean (nm)	154.1 ± 33.2	68	120.1 ± 36.0	42	103.9 ± 31.0	68	98.9 ± 27.2	71	75.0 ± 23.2	38	48.5 ± 29.6	39
Hole diameter—Range (nm)	105.3–229.7	68	64.6–202.9	42	49.2–177.5	68	49.1–165.4	71	36.3–141.1	38	19.1–136.7	39
Cell borders	Smooth curved		Smooth curved		Smooth curved		Smooth curved		Straight		Straight	

**Table 6:** Differences in the length of the epithelial cells at the surface of the spectacle of the nine study species from central to peripheral regions analysed from digital images obtained using scanning electron microscopy. The percentage differences and statistical significance are also provided.

Common name	Species	Cell length: Central	*n*	Cell length: Peripheral	*n*	% difference	Significance
Terrestrial							
Black tiger snake	*Notechis ater*	21.77 ± 1.99 µm	77	54.00 ± 10.47	68	+148	*p* < 0.00001
Coastal taipan	*Oxyuranus scutellatus*	19.13 ± 2.12 µm	76	50.62 ± 5.82 µm	57	+165	*p* < 0.00001
Eastern tiger snake	*Notechis scutatus*	23.40 ± 2.98 µm	62	45.55 ± 6.89 µm	67	+94.6	*p* < 0.00001
Mexican moccasin	*Agkistrodon bilineatus*	29.04 ± 4.07 µm	53	30.63 ± 3.79 µm	81	+5.4	*p* = 0.024
Aquatic							
Bar‐bellied seasnake	*Hydrophis major*	31.54 ± 4.79 µm	61	40.95 ± 4.50 µm	58	+28.4	*p* < 0.00001
Black‐ringed sea snake	*Hydrelaps darwiniensis*	21.61 ± 1.69 µm	67	23.62 ± 2.21	67	+9.3	*p* < 0.00001
Elegant seasnake	*Hydrophis elegans*	28.09 ± 4.85 µm	118	31.25 ± 4.85 µm	95	+11.3	*p* < 0.00001
Olive‐brown seasnake	*Aipysurus laevis*	26.54 ± 2.80 µm	46	30.96 ± 3.46	56	+16.7	*p* < 0.00001
Stoke's seasnake	*Hydrophis stokesii*	23.45 ± 2.50 µm	57	34.28 ± 4.03 µm	63	+50.5	*p* < 0.00001

**Figure 7:**
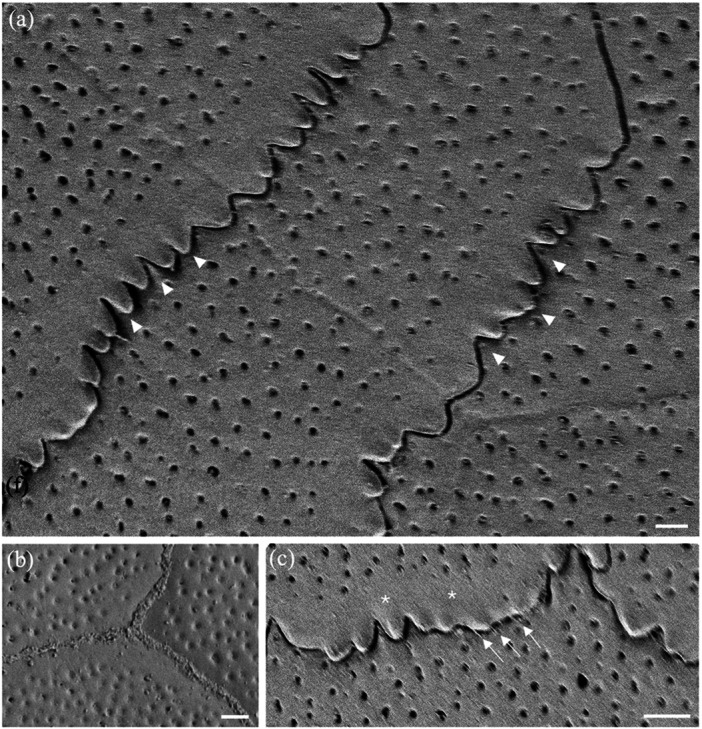
Surface ultrastructure of micro‐ornamentation and cell border connections. (a) High magnification of the elongated surface cells in the periphery of the spectacle in *Oxyuranus scutellatus* showing the sawtooth cell borders between cells (arrowheads). (b) High density of the microindentations over the surface of three adjacent epithelial cells in the central region of the spectacle in *Hydrelaps darwiniensis*, which possess curved borders. (c) Higher power scanning electron micrograph of the intersection of three epithelial cells in *O. scutellatus* showing a series of connections (arrows) between cells along their zigzag borders. Note also numerous microholes and micropits but not along the peripheral border of the upper cell (asterisks). Scale bars 0.5 µm (a–c).

As a way of comparing topographic changes in the density, shape and size of spectacular surface cells at a more granular level, a transect of the spectacle of two species of terrestrial snakes (*Notechis scutatus* and *Oxyuranus scutellatus*) and one species of aquatic snake (*Hydrophis elegans*) was undertaken at the level of the scanning electron microscope. Cell area, density, shape and length were all examined in each species within six topographic zones each between 200 and 250 µm in width from the periphery to the centre of the spectacle. All three species showed remarkably consistent changes (Tables 3, 4, 5). A broader comparison of cell length between central and peripheral regions of the spectacle, combining zones 1 and 2 and 5 and 6 is presented in Table 6. It shows a consistent increase in cell length between the centre and periphery from a 5.4% increase in *A. bilineatus* to a 165% increase in *O. scutellatus*. In some species, for example *O. scutellatus*, there are small attachments or bridges between the surface cells (Figures 5 and 7). These may represent desmosomes maintaining connections during ecdysis.

**Figure 8:**
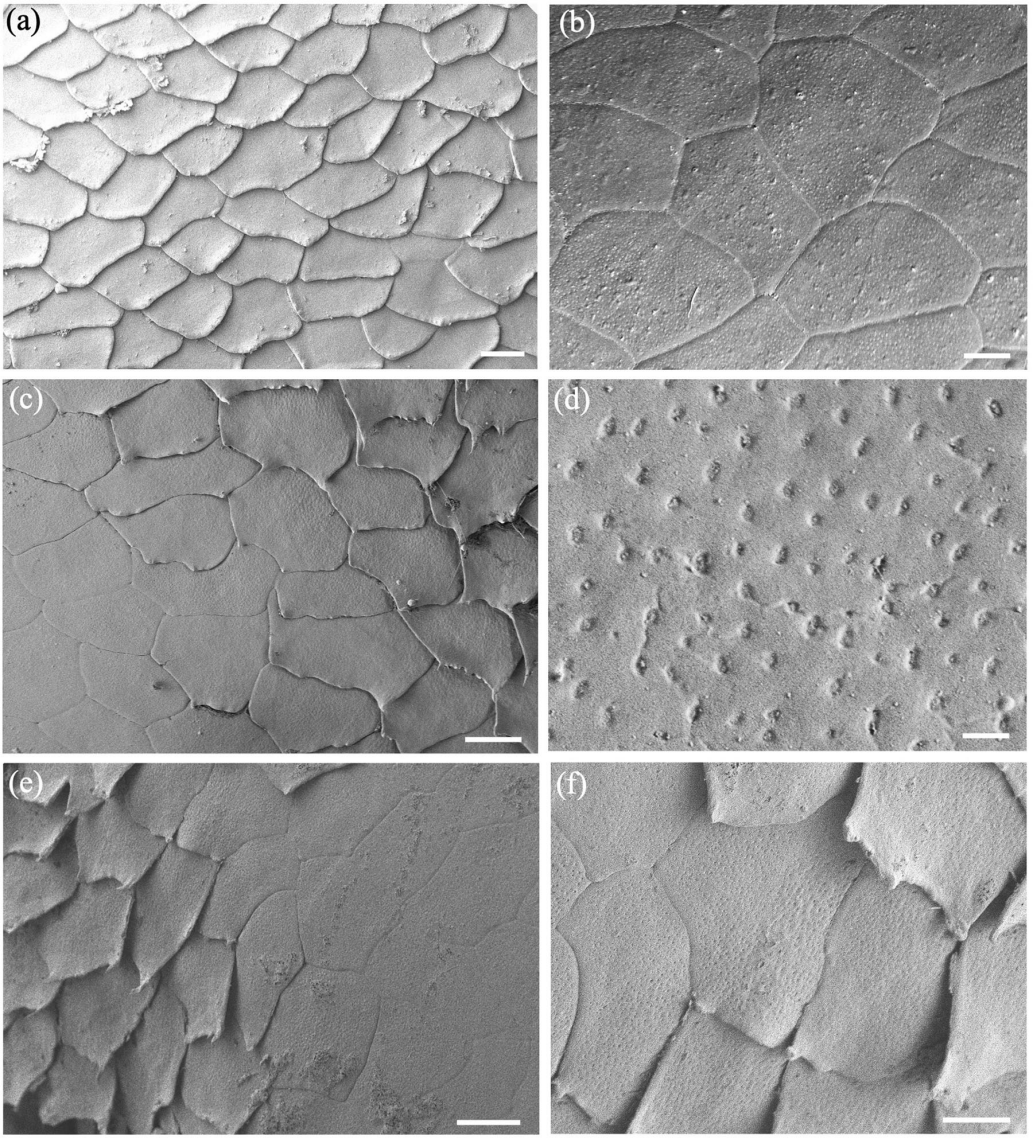
The transition of micro‐ornamentation from the spectacle to adjacent head scales. (a) Surface of the central region of the postocular scale in *Hydrophis major* showing the surfaces of the array of overlapping epithelial cells. Note the edge of each cell is slightly lifted on the side facing away from the spectacle. (b) Epithelial cells within the periphery of the supralabial scale of *Hydrophis elegans*. (c) Micrograph showing the transitional zone between the spectacle and the supralabial scale in *H. elegans*. Note the cells separate, develop pointed edges and lift or overlap at the extreme periphery of the spectacle, where the dermis invaginates to follow the curvature of the eyecup. (d) High power of the micropits (possibly filled with debris) that cover the supralabial scale of *H. elegans*. (e and f) Zone in the periphery of the spectacle of *H. elegans* showing the transition to the supralabial scale and the lifting of each epithelial cell. Scale bars 10 µm (a); 5 µm (b); 10 µm (c); 0.5 µm (d); 10 µm (e); 5 µm (f).

The cephalic scales of *H. elegans* and *H. major* adjacent to the peripheral spectacular cells are similar to the peripheral cells being elongated with smooth rounded cell borders, which transition into overlapping scales with the beginning of small extensions on the overlapping margin pointing away from the spectacle (Figure 8a,c,e,f). The cells of the spectacle never appear to be overlapping. These scales are densely populated with indentations, the majority of which appear to be micropits with a few scattered microholes (Figure 8d,f).

### 3.3 Micropits and Microholes

The spectacular cells of all nine species have small indentations in the surface (micropits) and/or microholes, which appear to penetrate the cell membrane into the cytoplasm of the cell (Figure 5d,e). In the Stoke's sea snake (*Hydrophis stokesii*), all the micropits are very shallow and none seem to penetrate the surface, that is, they are not actual holes, therefore, appear as indentations.

The diameters of these micropits and microholes are similar and have been grouped together and referred to as holes. In the centre of the spectacle, the mean diameter of these holes ranges from 42.39 ± 13.29 nm in the Olive sea snake, *Aipysurus laevis*, to 120.55 ± 43.93 nm in the Cantil viper, *Agkistrodon bilineatus* (Table 7). In the periphery, with the exception of Stoke's sea snake, *Hydrophis stokesii* and the black‐ringed sea snake, *Hydrelaps darwiniensis*, in which the apparent increases are not significant, the holes in all the other species are significantly larger than in the centre, ranging from 63.76 ± 20.18 nm in the Bar‐bellied sea snake, *Hydrophis major* to 182.60 ± 39.85 nm in *A. bilineatus* (Table 7). In several species, with a dense array of holes (or pits) in the cell surface, there is a wide, clear zone without holes along the rim of the central‐facing edge of the cell. This is particularly noticeable in *N. scutatus*, *N. ater* and *O. scutellatus* (Figures 5c,d,e and 7a,c). A similar absence of holes in a region of the cell surface is also found on the skin scales of some snake species (Campbell et al.^1999^).

**Table 7:** Differences in the diameter of holes in the epithelial cells at the surface of the spectacle of the nine study species from central to peripheral regions analysed from digital images obtained using scanning electron microscopy. The percentage differences and statistical significance are also provided.

Common name	Species	Hole diameter Central	*n*	Hole diameter Peripheral	*n*	% difference	Significance
Terrestrial							
Black tiger snake	*Notechis ater*	65.87 ± 19.33 nm	68	108.47 ± 87.81 nm	68	+64.7	*p* = 0.00015
Coastal taipan	*Oxyuranus scutellatus*	67.70 ± 20.09 nm	71	124.78 ± 32.99 nm	70	+84.3	*p* < 0.00001
Eastern tiger snake	*Notechis scutatus*	58.99 ± 19.43 nm	113	120.78 ± 29.84 nm	72	+104.7	*p* < 0.00001
Mexican moccasin	*Agkistrodon bilineatus*	120.55 ± 43.93 nm	229	182.60 ± 39.85 nm	186	+51.5	*p* < 0.00001
Aquatic							
Bar‐bellied seasnake	*Hydrophis major*	55.23 ± 14.38 nm	70	63.76 ± 20.18 nm	93	+15.5	*p* = 0.0030
Black‐ringed sea snake	*Hydrelaps darwiniensis*	62.04 ± 10.52 nm	54	68.51 ± 23.96 nm	78	+10.4	*p* = 0.0648
Elegant seasnake	*Hydrophis elegans*	62.34 ± 30.10 nm	78	141.12 ± 37.95 nm	110	+163	*p* < 0.00001
Olive‐brown seasnake	*Aipysurus laevis*	42.39 ± 13.29 nm	70	109.6 ± 24.62 nm	50	+159	*p* < 0.00001
Stoke's seasnake	*Hydrophis stokesii*	97.70 ± 19.44 nm	34	104 29 ± 23.42 nm	31	+6.8	*p* = 0.2197

**Figure 9:**
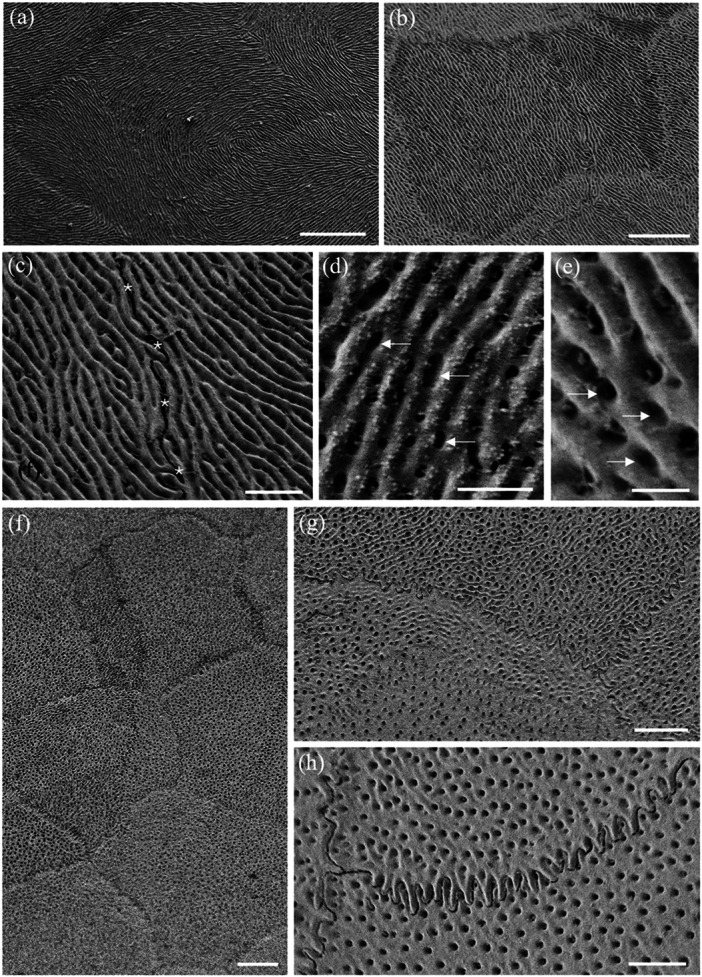
Changes of surface micro‐ornamentation in the spectacle of *Agkistrodon bilineatus* from centre to periphery. (a) Fingerprint‐like pattern of microridges over the surface of several epithelial cells. (b) Pattern of microridges over the surface of a number of epithelial cells in the centre of the spectacle. Note some microridges extend almost the diameter of an entire cell, while others are relatively short and branch multiple times. The orientation of the microridges is somewhat aligned (parallel) but where the orientation and/or height of the microridges changes, light and dark regions can be differentiated and sometimes within a single cell. (c) Close up of the complex of microridges away from the central region of the spectacle. Asterisks indicate the junction of two epithelial cells with fine attachments between the cells. (d and e) Close up of a parallel microridge pattern. Note the unevenness of the ridge surface and the elongation of the microholes between the raised struts of each ridge (arrows). (f) Lower power of several epithelial cells with a paucity of smaller microridges and high numbers of microholes. (g) Higher power of the transitional zone of the spectacle, where microridges decrease in size and density even within a single epithelial cell. (h) High power electron micrograph showing numerous microholes but only slightly raised microridges in some areas that are otherwise smooth. Note the zigzag cell borders. Scale bars 5 µm (a, b); 1 µm (c); 0.5 µm (d); 0.4 µm (e); 5 µm (f); 3 µm (g); 2 µm (h).

Both the density and the ratio of micropits to microholes vary greatly among the species studied. In addition, they vary significantly from cell to cell, making it almost impossible to assess accurately. For example, the polygonal cells in the periphery of the spectacle of the Cantil viper, *A. bilineatus*, and the elongated cells in the periphery of the Coastal taipan, *O. scutellatus*, and the Eastern tiger snake, *N. scutatus*, are covered almost entirely with what appear to be microholes, with micropits less common (Figure 7a,c). The situation is similar with only holes between the microridges in the spectacle of *A. bilineatus* (Figure 9, see below). In contrast, in Stoke's sea snake, *Hydrophis stokesii*, all the indentations are very shallow and none seem to penetrate the surface to form holes. Micropits also predominate in the mid‐periphery of the spectacle of *Hydrelaps darwiniensis* (Figure 7b) and *Hydrophis elegans*, while nearer the centre of the spectacle in *H. darwiniensis*, microholes are more prevalent. Significant cell‐to‐cell (between neighbouring cells) variations are found in *O. scutellatus* (Figures 5e and 7a,c). There was no significant difference between the size or the distribution of the microholes or micropits between the terrestrial and aquatic species (Table 7).

### 3.4 Microridges

**Figure 10:**
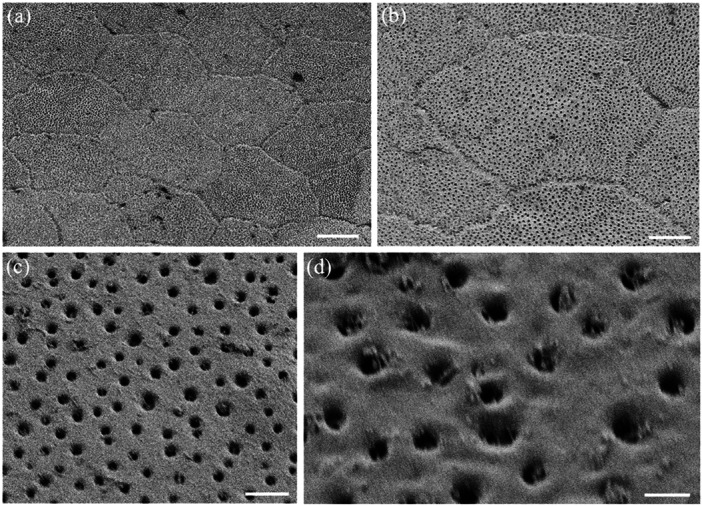
Changes of surface micro‐ornamentation in the peripheral region of the spectacle of *Agkistrodon bilineatus*. (a) Low power of the epithelial cell array showing the lack of any complexes of microridges but numerous microholes. (b) Higher power of a single epithelial cell surrounded by six other cells showing the variation in cell border types, which range from a zigzag pattern to irregular. (c) High power of the smooth surface of peripheral epithelial cells showing the absence of microridges and numerous, circular microholes. (d) High power of the smooth surface of the spectacular epithelial cells showing numerous microholes, each bordered by putative strands of mucus or debris. Scale bars 10 µm (a); 5 µm (b); 1 µm (c); 0.5 µm (d).

Microridges are found on the superficial cells of the spectacle of only one of the nine species examined, namely the terrestrial Cantil viper, *Agkistrodon bilineatus*. These microridges are 138.4 ± 28.2 nm wide in the centre, where the microridges are relatively straight and parallel, and 143.1 ± 19.1 nm wide in the periphery, where the microridges form more complex patterns (Figures 9 and 10). The difference in thickness is not significant (*p *= 0.117). The density of microridges decreases towards the periphery, where epithelial cells located midway between the centre and the periphery show intracellular transitions, which give way to peripheral cells without any ridges and a smooth surface (Figure 10). Microholes are present in between the microridges in this species although, where the holes in species without ridges are always round, the holes between the ridges are oval (compare Figure 9e with Figure 10c,d).

## 4 Discussion

### 4.1 Centro‐Peripheral Changes in Cell Density, Size and Shape

In the absence of moveable eyelids, the snake cornea is protected by a spectacle (Neher 1935; Walls 1940). Neher (1935) reported that the spectacle or “Brille” in front of the cornea allows the snake to keep its eyes open at all times to detect food, friend or foe. Walls (1940) claimed the universality of a tertiary spectacle in all snakes and defined it as distinctly extraocular, overlying a complete eyeball. Later, Walls (1942) described the eyes of snakes, including the spectacle, as thoroughly standardised in structure, although he also states that the peculiarities are numerous and great and that when the spectacle becomes dull and scratched, it is beneficial that it is shed and replaced.

The structure of the snake cornea and spectacle has been described by many authors based on observations using light microscopy (Walls 1942; Da Silva et al. 2014, 2016; Lauridsen et al. 2014), ultrasound (Lauridsen et al. 2014) and optical coherence tomography (Rival et al. 2015; Tusler et al. 2015; Da Silva et al. 2019). However, a few investigators have used transmission electron microscopy (TEM) to describe the morphology of the snake spectacle (Da Silva et al. 2014, 2016). In the Ball python, *Python regius*, Da Silva et al. (2014) examined the spectacle using light microscopy and TEM and found that the surface of the spectacle is covered by an outer epithelium comprised of flat basal cells overlaid by keratin. There appears to be only one publication (Campbell et al. 1999) that mentions the external surface of a snake spectacle using scanning electron microscopy (SEM).

### 4.2 Implications of Topographic Changes

With the exception of the terrestrial Eastern tiger snake, *Notechis scutatus*, the cell densities of the superficial layer of the snake spectacles are similar to those of the cornea of most terrestrial species, for example, among the Reptilia: the Crocodile (*Crocodilis porosus*, 2283 ± 824 cells/mm^2^), the Loggerhead turtle (*Caretta caretta*, 4191 ± 1672 cells/mm^2^) and the Ornate lizard (*Ctenophorus ornatus*, 5095 ± 1746 cells/mm^2^) (Collin and Collin 2000a); the Amphibia: the Salamander (*Ambystoma mexicanum*, 2918 ± 1753 cells/mm^2^) and the African clawed toad (*Xenopus laevis*, 7095 ± 3470 cells/mm^2^) (Collin and Collin 2006) and the Mammalia: human (*Homo sapiens*, 2525 cells/mm^2^) (Lemp and Gold 1986) and New Zealand rabbit, *Oryctolagus cuniculus* (1898 cells/mm^2^) (Doughty 1990). A comparison of the density of the outer surface cells of the spectacle and cornea is considered appropriate because both have similarities of structure and both represent the only surface or component of the visual system that is in direct contact with the environment. The cell densities of the snake spectacles in this study are considerably less than the corneas of many aquatic species, particularly members of the Osteichthyes, among which the cell densities range from 5999 cells/mm^2^ in the Black bream (*Acanthopagrus butcheri*) to 29,348 cells/mm^2^ in the Dover sole (*Microstomus pacificus*) (Collin and Collin 2000a, 2006).

In all species except *A. bilineatus* and *H. darwiniensis*, in which the differences were not significant (Table 1), the surface cell densities were greater in the centre of the spectacle than in the periphery. This is consistent with findings for the cornea of the Sandlance, *Limnichthyes fasciatus* (Collin and Collin 1988), the Dutch belt rabbit, *O. cuniculus* (Doughty and Fong 1992; Doughty 2008) and humans (Lemp 1989; Mathers and Lemp 1992), although Zheng et al. (2016) did not observe any difference in cell density in the human cornea using in vivo confocal microscopy. In the Little penguin (*Eudyptula minor*), the peripheral corneal cell density is significantly greater than in the centre, with a difference of up to 66.6% (Collin and Collin 2021b).

Thoft and Friend (1983) presented a theory for the high concentration of epithelial cells in the centre of the human cornea, suggesting that there is a continual centripetal migration as well as proliferation and desquamation. Lemp and Mathers (1989) claimed that there is preferential loss of surface cells by exfoliation from the central apex secondary to the shearing forces of the upper lid. However, this explanation cannot apply to the snakes in the absence of eyelids.

The occurrence of very long circumferentially‐oriented peripheral cells in the spectacle of the Australian land elapids, that is, the Coastal taipan, *Oxyuranus scutellatus*, the Black tiger snake, *Notechis ater* and the Eastern tiger snake, *N. scutatus* appears to be unique to reptiles. Similar cells are present in the skin of snakes and is referred to as ‘wide’ cells by Arrigo et al. (2019). That study examined 353 shed snake skins from numerous zoos and institutes around the world and found ‘wide’ cells, defined as ‘more than twice wider than long’, were present in 205 species, although this group contained a variety of cell shapes. Similarly‐shaped long cells have been described by other authors in the skin of the common boa, *Boa constrictor* (Amemiya et al. 1995), common European adder, *Vipera berus* (Hazel et al. 1999), goggle‐eyes worm snake, *Leptotyphlops macrolepi*s, Ternetz's blind snake, *Liotyphlops ternetzii* (Gower 2003) and Ball python, *Python regius* (Toro et al. 2021). However, long cells have been observed in the spectacle of the shed skin of only one species of snake, the Carpet python, *Morelia spilotes* (Campbell et al. 1999).

Some of the epithelial cells have cell borders with numerous interdigitations (zigzags), which are particularly marked on the circumferential sides, but not the ends, of the long spectacular cells in the periphery of the Black tiger snake, *Notechis ater* and the Coastal taipan, *Oxyuranus scutellatus*. These interdigitations may be regarded as a form of cell adhesion (Shelburne and Scroggs 1994) and may indicate a need for strong attachment in this region of the spectacle. A similar sign of possible tension was seen in some of the cells of *O. scutellatus*, where there is a slight separation of the cells with small attachments remaining, possibly indicating the presence of desmosomes. Desmosomes were also confirmed linking the central cells in the cephalic scales using transmission electron microscopy in the Olive seasnake, *A. laevis* by Crowe‐Riddell et al. (2019).

These interdigitations are quite distinct from the digitations found on the skin scales of snakes, which are up to 10 µm in length (Arrigo et al. 2019) and do not represent cell‐to‐cell contact. Those digitations give the snake skin its anisotropic properties with low friction for forward motion and high friction for backward movement (Hazel et al. 1999; Arnold 2002; Baum et al. 2014).

### 4.3 The Presence of Micropits and Microholes

Many authors have described indentations, pits or micropits in the scales of the skin of many snake species (Hazel et al. 1999; Campbell et al. 1999; Arnold 2002; Abdel‐Aal et al. 2010; Spinner et al. 2013, 2014; Toro et al. 2021). After examining several species, Price and Kelly (1989) found the width of these pits to be 0.5 µm. Arrigo et al. (2019) found holes in the shed skin scales in 128 of the 332 snake species they examined; however, this categorisation did not include many other species which had holes in addition to other characteristics.

Micropits and/or microholes are present on the surface of the spectacles of all nine species examined, whether or not microridges were present. This is true for both ‘straight’ and ‘labyrinthine’ microridges or ‘channels’ (as designated by Arrigo et al. 2019), which we found present in the same individual, namely the Cantil viper, *Agkistrodon bilineatus*, a member of the Viperidae. This compares with the findings of Arrigo et al. (2019), who found only 59 of the 328 shed snake skins they examined as having ‘smooth’ scales, namely, without holes, micropits or channels (microridges). Gower (2003) found that in 23 of the 27 fossorial species studied, in which the snake skin had no or very small pores/pits, the Oberhäutchen appears to be unperforated, when viewed at magnifications up to 10,000 times using scanning electron microscopy, that is, he refers to pores/pits but never mentions holes.

Some of these differences may be due to the preservation methods employed or the stage of skin shedding, which is unknown. We preserved our specimens in either 4% paraformaldehyde or Karnovsky's fixative (2% paraformaldehyde and 2.5% glutaraldehyde) immediately after capture and euthanasia. Shed snake skins from many sources were examined by Arrigo et al. (2019), whereas Gower (2003) studied museum specimens stored in methylated spirits and ethanol. Therefore, the preservation methods may influence the relationship between the nanomorphology of the scales of the skin and the spectacle.

The mean diameter of the micropits and microholes in the peripheral spectacle of the nine study species (mean 113.7 nm, with a range of 63.8–182.6 nm) is slightly smaller than the micropits found in the peripheral spectacle of the Australian Carpet python, *Morelia spilotes*, namely 179 ± 45 nm (Campbell et al. 1999), however, those in the centre of the spectacle in the current series (mean 70.3 nm, with a range of 42.4–120.6 nm) are much smaller. In the scales of the skin, the size of the micropits varies with some species having a similar diameter to those found in the spectacle (this study), that is, 200–250 nm in the Ball python, *Python regius* (Abdel‐Aal et al. 2010) or much larger diameters, with micropits measuring at up to 0.5 µm in the Striped crayfish snake, *Liodytes alleni* and Hampton's slug snake, *Pareas hamptoni* and 1.0–2.0 µm in the Amethystine python, *Liases amethystinus* and the common slug snake, *Pareas monticola* (Price and Kelly 1989). Neither of these studies on snake scales makes any mention of holes or microholes.

Campbell et al. (1999) found the depth of the micropits in the spectacle of the Australian carpet python, *Morelia spilotes*, to be 6 ± 3 nm but make no mention of holes. These pore‐like depressions are shallower than in other areas, such as the skin, and do not form channels to any underlying lipid‐secreting organs in the epidermis or dermis (Campbell et al. 1999).

### 4.4 The Structure and Function of Micropits and Microholes

Many possible functions have been presented for the presence of micropits and holes in the skin of snakes and many of these may or may not apply to the micropits and holes present in the spectacle. From their studies of the Carpet python, *Morelia spilotes*, Campbell et al. (1999) suggested that the arrays of micropits and plate surface structures over the scales and ocular spectacle, act as spectral filters or anti‐reflective coatings with respect to incident electromagnetic radiation. Amemiya et al. (1995) speculated that, at least in the scales of the pit organs, the micropits (pores) might act as natural infrared detectors by reflecting away shorter wavelengths in the visible spectrum, while allowing longer infrared wavelengths to pass through. Pores are present on the surface epithelium of the infrared receptors of members of the Boidae and the subfamily Crotalinae (within the family Viperidae), where the array appears to reflect and diffuse visible radiation that might interfere with the target stimulus, that is, infrared radiation (Amemiya et al. 1995).

The closely‐packed pores or pits might also serve to increase non‐wettability and therefore increase the dirt‐shedding ability of the surface (Gans and Baic 1977; Gower 2003). This is supported by Arrigo et al. (2019), who found that the body scales of some fossorial uropeltid ‘sunbeam’ snakes bear regular nanoscopic depressions and digitations that confer spectacular hydrophobicity and iridescence. In contrast, Arnold (2002) claimed that, at least in the skin of lizards, the pitted surfaces might be more prone to hold dirt.

Andonov et al. (2020) analysed what they call ‘the skin secretions’ on the surface of *Viper ammodytes* and found 59 compounds, of which six were ketones. They speculate that the hydrocarbons are contaminants from the environment but do not comment on the source of the other substances. A lipid coating is present on the surface scales of the Californian kingsnake, *Lampropeltis californiae*, which is thought to provide both lubrication and protection (Baio et al. 2015). These extracellular epidermal lipids appear to serve as the major barrier across the snake skin (Roberts and Lillywhite 1980) but may also act as pheromones for chemoreceptive recognition of a sexual partner in the turtle‐headed sea snake, *Emydocephalus annulatus* (Shine 2005). Toro et al. (2021) claim that micropits are commonly linked with the enhancement of lubrication efficiency of these surfaces and it is suggested that the epidermal pores provide the only access for the lipids to the skin surface (Chiasson et al. 1989; Chiasson and Lowe 1989). In addition, it has also been suggested that micropits may be part of a system of continuous micropores penetrating through the snake skin and may serve as a delivery system for the lubrication/anti‐adhesive lipid mixture that provides for boundary lubrication of snake skins and serves to control wettability and permeability (Hazel et al. 1999). Similar functions may be attributed to the micropits/microholes in the spectacle. According to Chiasson and Lowe (1989), the pores may act as fasteners that secure the outer keratinised epidermal layer to the inner layer until the skin is removed through shedding or ecdysis.

The presence of holes in a primary spectacle has been reported for the Pouched lamprey, *Geotria australis* (Collin and Collin 2000a, 2006), the Shorthead lamprey, *Mordacia morda)* (Collin and Collin 2006) and the ammocoete stage (Dickson and Graves 1981), but not the adult stage, of the Sea lamprey, *Petromyzon marinus* (van Horn et al. 1969; Pederson et al. 1971; Dickson and Graves 1981). Surface holes also occur in the pre‐metamorphic axolotl, *Ambystoma mexicanum*, in which there is a spectacle (Collin and Collin 2000b, 2006, 2021a) and where they represent a means of accessing water and oxygen into the cornea and a route for the release of mucus onto the corneal (spectacular) surface, as a way of ensuring a smooth and protective tear film. In addition, holes are associated with burrowing in this species and they have almost completely disappeared in the post‐metamorphic stage (Collin and Collin 2021a) in the absence of a spectacle, when burrowing is less likely to occur.

Microholes are present in the superficial layer of the cornea of numerous species from various classes of vertebrates, including the Chondrichthyes, for example, the Black shark, *Dalatias licha* (Collin and Collin 2006) and Osteichthyes, for example, the Australian lungfish, *Neoceradotus forsteri* and the salamanderfish, *Lepidogalaxias salamandroides* (Collin and Collin 2000b). In *L. salamandroides*, the holes (571 nm in diameter) in the cornea are outlets for goblet cells in the surface epithelium (Collin and Collin 1996), while in the spectacle of the Pouched lamprey, *Geotria australis* (Collin et al. 2021) and the Shorthead lamprey, *Mordacia mordax* (Collin et al. 2022) and in the cornea of the Australian lungfish, *Neoceratodus forsteri* (Collin et al. 2023), the holes are the openings to long (up to around 4 µm) cylindrical channels, through which mucous vesicles are passed from the cytoplasm to the corneal surface to protect the cornea against dehydration, when exposed to air and damage while burrowing. In *G. australis*, there is a significant inverse relationship between the diameter of the spectacular epithelial surface holes and cell size, in that the smallest epithelial cells had the largest holes and the larger cells had smaller holes with the largest cells having no holes (Collin et al. 2021). This was not apparent in the *M. mordax* (Collin et al. 2022) but was present in the pre‐metamorphic stage in the axolotl, *A. mexicanum* (Collin and Collin 2021a). Such a relationship does suggest that the development of holes is a dynamic process associated with the epithelial growth; however, this was not found in the spectacle of any of the snakes examined in this study.

The markedly varied distribution of micropits and microholes between species and even among spectacular cells of the same species in this study raises the possibility that this is also a dynamic situation, in which micropits may represent a stage in the development or regression of microholes, similar to that seen in the Pouched lamprey, *G. australis*, where the hole size changes with the development of the cell (Collin et al. 2021). In both *G. australis* and *M. mordax* (Collin et al. 2022), the holes are channels for the release of mucus and perhaps other substances.

The microholes appear similar to the holes seen in vascular, lymphatic and corneal endothelia, where pinocytotic vesicles, which are involved in the transport of various molecules across the endothelium, break through the cell membrane. These vesicles are typically 75.7 ± 1.53 nm in maximum diameter in various endothelia (Casley‐Smith 1969) or between 60 and 80 nm, depending upon the endothelial type and fixation procedures (Mazzone and Kornblau 1981). Our findings of mean diameters of the micropits and microholes for the nine species (e.g., central 70.3 nm, peripheral 113.8 nm and a mean of 93.7 nm of both central and peripheral regions, Table 7) are similar to the diameter of the pinocytotic vesicles. A similar situation occurs in the carp, *Cyprinus carpio*, where mucus is secreted from the surface of the oral mucosal epithelium in between the microridges (Uehara et al. 1988). Just as the holes in many of these species form outlets for mucus or other compounds, a dynamic system of micropits/microholes would facilitate a system of continuous micropores penetrating the snake skin, as suggested by Hazel et al. (1999) for the delivery of various substances to the snake skin surface.

### 4.5 The Structure and Function of Microridges

Microridges are present in only one of our nine species, that is, in the Cantil viper, *Agkistrodon bilineatus*, which is distantly related to the other elapid species sampled in this study. Arrigo et al. (2019) found microridges (channels) on shed skin scales of 135 of the 353 snake species they studied and, of these, only 10 were described as having a ‘labyrinthine’ pattern similar to the more peripheral cells in *A. bilineatus*. In fact, 7 of the 10 in that series were also members of the Viperidae family. In addition, the spectacle of *A. bilineatus* had a “straight channel” pattern, similar to that described by Arrigo et al. (2019) for the central region of skin scales. Similar patterns (reticulate and canaliculate) also have been described in the skin scales in a range of species within the genus *Agkistrodon* by Chiasson et al. (1989). Microridges have been reported over the surface of the cornea of the ornate lizard, *Ctenophorus ornatus*, which has evolved moveable eyelids in favour of a spectacle (Collin and Collin 2000b). Similar microridges are present on the surface of the spectacle of the agnathan Pouched lamprey, *G. australis* (Collin et al. 2021) and on the cornea of many bony fishes (Osteichthyes), for example, the scup, *Stenotomus chrysops* (Harding et al. 1974) and the four‐eyed fish, *Anableps anableps* (Simmich et al. 2012). Among 14 different species of teleosts, the widths of the microridges ranged from 119 nm in the West Australian seahorse, *Hippocampus augustus* to 250 nm in the sandlance, *Limnichthyes fasciatus*, with a mean of 171.6 ± 42.7 nm (Collin and Collin 1997, 2000b, 2006). These microridges vary greatly in their length and in the complex patterns they display. One representative of the Aves, namely, the Little penguin, *Eudyptula minor* and one species of mammal, the Australian koala, *Phascolarctos cinereus* also have microridges on their corneal surfaces with widths of 87 ± 13 nm and 103 ± 14 nm, respectively (Collin and Collin 2006).

These microridges contain numerous filaments, the majority of which are intermediate and actin filaments, while at the bottom of the microridge there is a plexus of keratin filaments. These filaments play an important role in the maintenance of the structure of the microridge (Uehara et al. 1991). Similar to pits and micropits, the microridges on the skin of snakes are thought to contribute to the level of hydrophobicity and dirt shedding (Gower 2003; Spinner et al. 2014; Arrigo et al. 2019) and possibly the amount and intensity of shine (Arnold 2002). As early as 1976; Sperry and Wassersug (1976) suggested that the microridges help to hold a protective coating of mucus to the epithelium and the complex curved or whorled arrangement of microridges appears to facilitate the spread of mucus away from goblet cells. This mucus coating protects the surface of the corneal epithelium, especially as a barrier to pathogens, helps maintain hydration of the ocular surface and is an important component of the corneal tear film (Hodges and Dartt 2013).

Price and Kelly (1989) reported that in the skin of *Vipera berus*, its tiny surface pits tend to coalesce into ‘grooves and vermiculations’, presumably referring to the microridges of the type described in this study. However, neither the description nor the figures indicate whether any of the pits penetrate into the cell. A similar theory was presented by Arrigo et al. (2019), namely, that the distribution of shapes and structures classified as ‘holes’ and ‘channels’ in the skin of snakes, suggests the existence of a continuum between these two states and that channels simply evolved through the elongation of holes. However, holes are present between microridges in the spectacle of the Cantil viper, *A. bilineatus* and microridges exist on the corneas, spectacles and scales of many other species, such as agnathans and teleosts, with or without holes (Collin and Collin 2006).

Caprette et al. (2004) claimed that snake eyes exhibit similarities to those of aquatic vertebrates and suggest that the early evolution of snakes occurred in aquatic environments. Although this is consistent with our findings because of the appearance and prevalence of microridges in so many representatives of the Osteichthyes, the finding of microridges only in one (terrestrial) species appears to contradict this assertion. Therefore, the spectacle of more species needs to be examined but, whatever their origin, we believe that the microridges do not develop in the form of channels resulting from the fusion of surface holes or indentations, as suggested by Arrigo et al. (2019). It is possible that the microridges in *A. bilineatus* are an adaptation in the Viperidae that alters the texture of the spectacle, as described for the skin scales, that is used for camouflage for its ‘sit and wait’ hunting strategy (Spinner et al. 2013).

### 4.6 Comparison of Terrestrial and Aquatic Findings

As only one species of the nine studied possesses microridges and all of our snakes have micropits/microholes, no differences between terrestrial and aquatic species were discernible. Arrigo et al. (2019) suggested that phylogeny constrains nanograting morphology, whereas life habits (aquatic, terrestrial etc.) do not significantly co‐vary with any of the nanomorphological characters they investigated.

The averages of the mean cell densities for the four species of terrestrial snakes (4606 cells per mm^2^) were compared with the mean cell densities of the five aquatic species (3093 cells per mm^2^) from Table 2. Although the cell density is greater in the terrestrial species, the number of species examined is too small to assume significance. In previous studies (Collin and Collin 2000b; Collin and Collin 2001), the mean corneal epithelial cell density of 17 aquatic species (within the Agnatha, Chondrichthyes and Osteichthyes) was found to be 15,957 cells per mm^2^ compared with seven aerial species (within the Aves) with 8236 cells per mm^2^ and 13 terrestrial species (within the Amphibia, Reptilia and Mammalia) with 3485 cells per mm^2^. In the ‘four‐eyed’ fish *Anableps anableps*, the aquatic cornea has a significantly greater epithelial density than the exposed cornea, which is consistent with terrestrial species (Simmich et al. 2012). In both the central and peripheral surface epithelial cells of the spectacles of our nine species, the mean cell densities are numerically greater in the terrestrial snakes than in the aquatic species; however, these findings are not statistically significant.

## 5 Conclusions

The surface of the spectacle comprises a layer of cornified tissue (the Oberhäutchen) that reflects the stage of ecdysis that is continuous with the circumocular head scales. A comparison of the cell topography and surface micro‐ornamentation of the spectacles of representative members of land and sea snakes primarily from the Elapidae family reveals that the spectacle and scales share many common nanostructures but with some important differences. These include pronounced topographic changes in the size, density and shape of surface epithelial cells from central to peripheral regions of the spectacle that may reflect developmental processes and levels of adhesion. The spectacular cells of all nine species have small indentations in the surface (micropits) and/or microholes, which vary in size and may penetrate the cell membrane into the cytoplasm and may be part of a dynamic transport process and/or lubrication to control wettability and permeability. Further research is necessary to investigate interspecific differences in surface lipid levels and the ultrastructure of the outer layers of the spectacle and how this may affect vision. Microridges are present on only one species of land snake (a member of the Viperidae), which may contribute to the levels of hydrophobicity, dirt shedding and iridescence and facilitate the spread of mucus to maintain hydration and the integrity of the tear film. The presence of microridges in only one terrestrial species may be the result of the need to camouflage the spectacle, an adaptation which is vital for their “sit and wait” hunting strategy. The micro‐ornamentation of the spectacle in snakes appears to provide a range of functional properties, while still allowing a clear optical pathway for vision.

## Author Contributions

**H. Barry Collin:** conceptualization, investigation, writing – original draft, methodology, formal analysis, data curation, validation, visualization, writing – review and editing. **Myoung Hoon Ha:** methodology, visualization, investigation, validation, writing – review and editing. **Alizee Wagner:** investigation, methodology, visualization, validation, writing – review and editing. **Megan Folwell:** resources, methodology. **Nathan Dunstan:** methodology, resources. **Jenna Crowe‐Riddell:** investigation, methodology, visualization, resources, writing – review and editing, project administration. **Shaun P. Collin:** conceptualization, investigation, funding acquisition, writing – review and editing, methodology, validation, visualization, resources, project administration.

## Conflicts of Interest

The authors declare no conflicts of interest.

## Peer Review

The peer review history for this article is available at https://www.webofscience.com/api/gateway/wos/peer-review/10.1002/jmor.70084.

## Data Availability

The data that support the findings of this study are available on request from the corresponding author. The data are not publicly available due to privacy or ethical restrictions.
